# Continuous Circulation of Hepatitis E and A Viruses During COVID-19 Pandemic Lockdowns in Munich, Germany—Experience from Three Years of Wastewater Surveillance

**DOI:** 10.3390/microorganisms13102379

**Published:** 2025-10-15

**Authors:** Jasmin Javanmardi, Mathias Schemmerer, Karina Wallrafen-Sam, Jessica Neusser, Raquel Rubio-Acero, Michael Hoelscher, Thomas Kletke, Bernhard Boehm, Michael Schneider, Elisabeth Waldeck, Martin Hoch, Merle M. Böhmer, Christof Geldmacher, Jan Hasenauer, Jürgen J. Wenzel, Andreas Wieser

**Affiliations:** 1 Institute of Infectious Diseases and Tropical Medicine, LMU University Hospital, LMU Munich, 80802 Munich, Germany; jasmin.javanmardi@med.uni-muenchen.de (J.J.); jessica.neusser@lgl.bayern.de (J.N.); raquel.rubio@med.uni-muenchen.de (R.R.-A.); michael.hoelscher@med.uni-muenchen.de (M.H.); christof.geldmacher@med.uni-muenchen.de (C.G.); 2 National Consultant Laboratory for HAV and HEV, Institute of Clinical Microbiology and Hygiene, University Medical Center Regensburg, 93053 Regensburg, Germany; mathias.schemmerer@klinik.uni-regensburg.de (M.S.); juergen.wenzel@klinik.uni-regensburg.de (J.J.W.); 3 Life & Medical Sciences Institute (LIMES), Bonn Center for Mathematical Life Sciences, University of Bonn, 53115 Bonn, Germany; karina.wallrafen@uni-bonn.de (K.W.-S.); jan.hasenauer@uni-bonn.de (J.H.); 4 Department Task Force Infectious Diseases, Bavarian Health and Food Safety Authority, 80636 Munich, Germany; 5 Immunology, Infection and Pandemic Research, Fraunhofer Institute for Translational Medicine and Pharmacology ITMP, 80799 Munich, Germany; 6 German Center for Infection Research (DZIF), Partner Site Munich, 80802 Munich, Germany; 7 Unit Global Health, Helmholtz Zentrum München, German Research Center for Environmental Health, 85764 Munich, Germany; 8 Munich Metropolitan Sewer Authority (MSE), 81671 Munich, Germany; thomas.kletke@muenchen.de (T.K.); bernhard.boehm@muenchen.de (B.B.); m.schneider@muenchen.de (M.S.); 9 Public Health Department Munich (GSR), 80335 Munich, Germany; elisabeth.waldeck@muenchen.de; 10 Task Force for Infectious Diseases (GI-TFI), State Institute for Health II, Bavarian Health and Food Safety Authority (LGL), 80636 Munich, Germany; martin.hoch@lgl.bayern.de (M.H.); merle.boehmer@lgl.bayern.de (M.M.B.); 11 Institute of Social Medicine and Health Systems Research, Otto-von-Guericke-University, 39120 Magdeburg, Germany; 12 Institute of Computational Biology, Helmholtz Zentrum München—German Research Center for Environmental Health, 80939 Neuherberg, Germany; 13 Max von Pettenkofer Institute of Hygiene and Medical Microbiology, Faculty of Medicine, LMU Munich, 80336 Munich, Germany

**Keywords:** hepatitis E, hepatitis A, orthohepevirus A, orthohepevirus E, sewage, lockdown, transmission, correlation, natural experiment

## Abstract

The COVID-19 pandemic has increased interest in wastewater-based epidemiology (WBE) as a reliable and cost-effective framework for monitoring the spread of microbes. However, WBE frameworks have rarely been applied to the study of fecal–oral transmissible diseases, except for poliomyelitis. Here, we investigated the presence of hepatitis A virus (HAV) and hepatitis E virus (HEV) in wastewater in Munich. We collected wastewater samples between July 2020 and November 2023. A total of 186 samples were processed using centrifugation and analyzed for HAV- and HEV-RNA using RT-qPCR. As a reference, we used notification data from clinically or laboratory-diagnosed hepatitis A and E cases. Lockdown stringency levels were derived from official documentation. Our results show that 87.6% of wastewater samples were positive for HEV at concentrations of 9.0 × 10^1^ to 2.5 × 10^5^ copies/L, while HAV was only detectable in 7.5% of the samples at viral loads of 4.6 × 10^1^ to 2.4 × 10^3^ copies/L. We also detected differences in HEV concentrations but not in case numbers when comparing lockdown and no-lockdown periods. This study covers all but the first lockdowns in Bavaria. We present a unique real-world dataset evaluating the impact of lockdown interventions on hepatitis A and E case numbers, as well as on the concentrations of HAV and HEV in wastewater. Person-to-person spread and eating out appear to have contributed to the transmission of HEV. In addition, the consistently high HEV concentrations in sewage support the findings of serological studies, indicating a substantial burden of undetected subclinical infections.

## 1. Introduction

During the COVID-19 pandemic, wastewater-based epidemiology (WBE) became popular as a surveillance tool for tracking the epidemic trends and genetic variants of SARS-CoV-2 in large populations [1]. WBE represents a cost-efficient approach with a reduced analytical workload, while maintaining population-level monitoring capabilities. Furthermore, it is independent of notification data and legal testing requirements for subjects, thus ensuring population representativeness. Beyond SARS-CoV-2, WBE has also been applied to monitor other key pathogens such as the Respiratory Syncytial Virus (RSV) and the Influenza virus [2]. There is growing interest in establishing continuous wastewater monitoring for a broader range of infectious pathogens.

The effectiveness of monitoring wastewater for pathogens depends on two factors: the high rate of pathogen shedding by infected individuals and the high resilience of pathogens to sewage. Fecal–oral transmitted pathogens inherently fulfill these requirements, demonstrating remarkable stability within the harsh sewage environment and shedding in large quantities [3]. Historically, poliovirus has been a prominent target within this group of pathogens, with surveillance programs consistently demonstrating highly sensitive viral detection [4]. Given their high fecal shedding rates and alimentary or fecal–oral transmission routes, hepatitis A virus (HAV) and hepatitis E virus (HEV) likewise emerge as compelling candidates for WBE-based surveillance. In recent years, studies have demonstrated the prevalence of HEV in different water matrices [5,6], affirming its general suitability for WBE. More recently, a few reports confirm this observation of HEV in untreated wastewater [7]. However, the correlation between wastewater HEV concentrations and reported clinical cases or living conditions remains limited or has not been performed at all [8].

This is particularly relevant given HEV’s worldwide distribution and the limited availability of vaccines, which are currently licensed solely in China and Pakistan [9,10]. Furthermore, HEV differs from HAV insofar as individuals can acquire multiple re-infections with HEV [11]. Although early reports focused on a smaller number of fatal cases in pregnant women, it is now evident that the majority of HEV infections remain asymptomatic or subclinical, thus evading clinical detection [12,13]. Consequently, many infected individuals, who are not cognizant of their condition, shed high concentrations of HEV over a period of several weeks. HEV genotype 3, predominantly found in Europe, has been identified as a circulating strain in domestic pigs and wild boars. Humans are primarily exposed via the consumption of undercooked pork or raw sausages containing pork products. In other regions of the world, HEV transmission occurs via the fecal–oral route, through contaminated water [14].

HAV is less common in Germany [15] and, although it does spread within the country via contaminated food [16,17], it is widely considered a traveler’s disease. The number of reported infection cases is approximately five times lower for HAV than for HEV [15]. It spreads from human to human via the direct fecal–oral route or through food processing [18]. Absolute numbers of infections with this acute hepatitis are also lower, because one infection confers lifelong immunity, as does the vaccine. Additionally, many at-risk individuals become infected in childhood, generating subsequent lifelong immunity. In children, the disease is often subclinical or only manifests as diarrhea without noticeable jaundice and is therefore never diagnosed [19,20]. Still, due to its potential to cause outbreaks, HAV might be a relevant target for longitudinal surveillance in wastewater.

**Figure 1:**
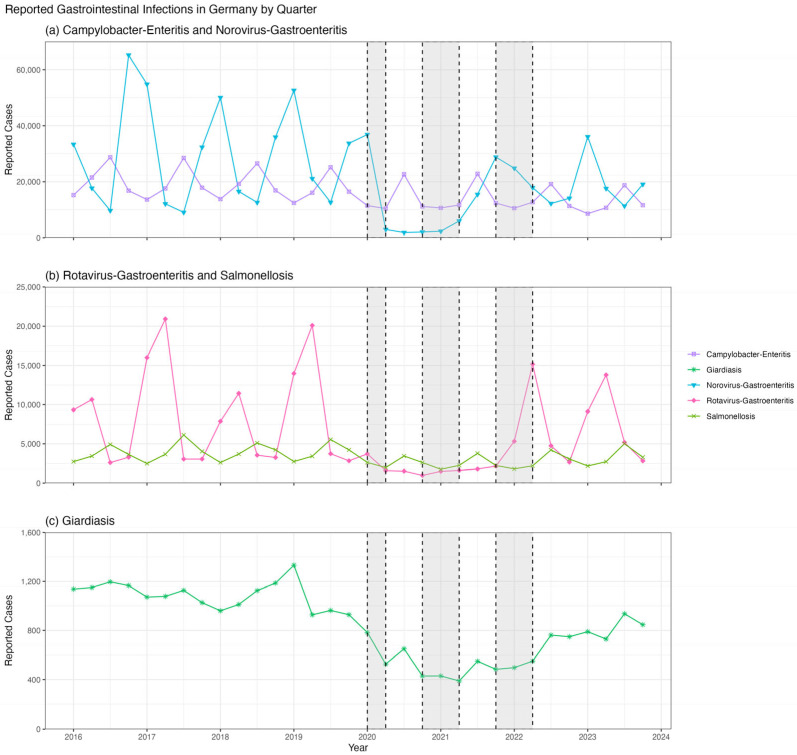
Reported cases of selected GI-infections in Germany between 2016 and 2023 according to the German Infection Protection Act (IfsG) § 7: reporting of all laboratory confirmed cases. Lockdown periods in Bavaria are highlighted within dotted boxes. During the lockdown years of 2020–2022, the number of cases reported for these selected GI diseases decreased overall. In non-restricted quarters, the reporting numbers of (**a**) campylobacter enteritis, norovirus-gastroenteritis (**b**) salmonellosis and rotavirus-gastroenteritis, and (**c**) giardiasis were higher than in lockdown periods [15]. School closures only occurred during the first two lockdown periods.

In this study, we present a longitudinal WBE study of HAV and HEV spanning approximately three years (from July 2020 to November 2023). This timeframe encompasses both periods of stringent SARS-CoV-2 lockdown measures—including forced closures of hospitality venues offering dine-in, food pick-up, or delivery options, as well as schools, and strict limits on gatherings—and the intervening and subsequent intervals with eased or no restrictions. The lockdowns resulted in a marked decrease in other fecal–oral diseases throughout Germany (Figure 1). Travel restrictions were also imposed, limiting the travel of individuals from Munich to areas where HAV or other HEV genotypes are endemic. We combine this data with the official notification data for both diseases within the Munich population to estimate the spread during lockdown and travel restrictions, and thereafter.

## 2. Materials and Methods

### 2.1. Wastewater Sampling

**Figure 2:**
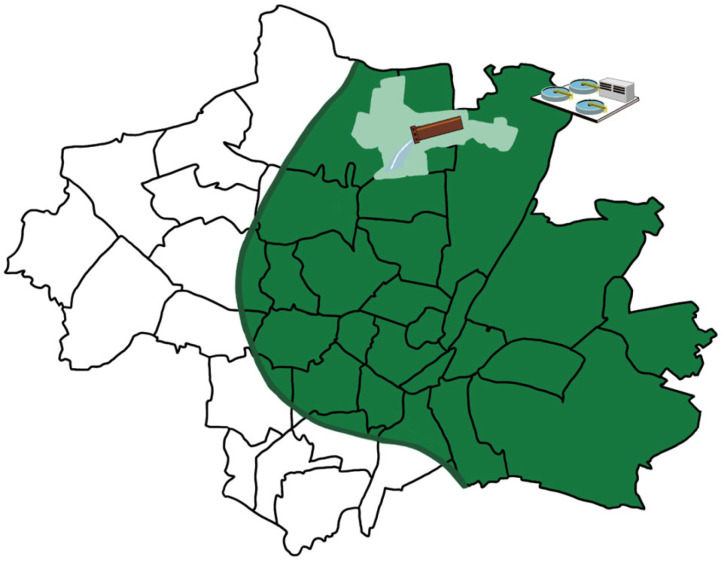
Map of the catchment areas of Schmidbartlanger and WWTP Gut Grosslappen. Light green shows the catchment area of Schmidbartlanger, for which we were provided with qualified spot samples. Dark green shows the catchment area of WWTP Gut Grosslappen, for which we were provided with 24-h compound samples.

Wastewater sampling was conducted as described previously from July 2020 to November 2023 in Munich, Germany [21]. For this study, we chose the area of Schmidbartlanger, which has a total of 66,914 permanent inhabitants. With a drainage area size of 670 ha and a maximum time of five hours from sink to sampling, this sampling site was chosen to be representative of the Munich population. The sampling time was chosen to reflect the morning flush surge. As of November 2021, we also analyzed a 24-h compound sample from the wastewater treatment plant (WWTP) Gut Grosslappen, located in the northern part of Munich, with a catchment area of about 961,207 permanent inhabitants. The areas are depicted in Figure 2. Sample transportation, processing, and storage were performed as previously described [21].

### 2.2. Comparison of Wastewater Concentration Methods

Prior to concentrating retrospective wastewater samples, we investigated different methods of concentration, including 1. Ultracentrifugation or 2. polyethylene glycol (PEG) precipitation, in combination with automated nucleic acid extraction using (a) TANBead Maelstrom 4810 (Taiwan Advanced Nanotech Inc., Taoyuan City, Taiwan) or (b) KingFisher Apex (Life Technologies GmbH, Darmstadt, Germany).

Water from a pond served as a negative reference, while effluent wastewater from the municipal WWTP was used as a matrix for spiking with virus stocks produced in cell culture. HAV genotype IB strain 18-35519 has been isolated in HuH-7 (passage 0; unpublished), while HEV genotype 3 (subtype 3c) strain 14-16753 has been isolated and passaged in PLC/PRF/5 (passage 3) [22]. A serial dilution of HAV and HEV was prepared and spiked into the water matrix. Following concentration, extraction, and RT-qPCR analysis, the loss factor associated with each step of the method was calculated. This method, utilizing approach 1a, exhibited the lowest overall loss factor. The limit of detection (LOD) and limit of quantification (LOQ) of the RT-qPCR protocol were determined using this spiking experiment. The LOD for HAV per RT-qPCR reaction is three copies, equivalent to 8.4 IU; for HEV, it is 14 copies, equivalent to 12.1 IU. LOQ ranges from the respective LOD up to 10 × 10^6^ copies per RT-qPCR reaction.

### 2.3. Virus Concentration and Nucleic Acid Extraction

Viruses in wastewater were concentrated from pre-filtered, freshly drawn, or frozen samples. Compared to our previous protocol using fresh wastewater (WW) [21], when frozen, we used the double-volume samples. In brief, 2 × 50 mL of frozen WW was centrifuged (Corning Science Mexico S.A. de C.V., Reynosa, Mexico) at 3000× *g*, 4 °C for 20 min to remove debris and bacteria. Then, 2 × 38 mL of the supernatant was transferred to an ultracentrifuge tube (Thermo Scientific Nalgene, Schwerte, Germany) and subsequently centrifuged at 26,000× *g*, 4 °C for 1 h. The pellet from the first tube was resuspended in 300 µL of nuclease-free water and transferred to the corresponding second tube. The pellet in the second tube was then resuspended.

RNA extraction was performed using an automated nucleic acid extraction system, the TANBead Maelstrom 4810 (Taiwan Advanced Nanotech Inc.). The TANBead Optipure Viral Auto Tube M665S46 Isolation Kit (LabConsulting GmbH, Vienna, Austria) was used for magnetic-based RNA-isolation. Briefly, the resuspended pellet was combined with 10 µL proteinase K, which was provided with the kit, and added to the extraction tube. The pre-programmed “667 rapid” extraction program was initiated on the instrument. Whole nucleic acids were eluted in 70 µL of the elution buffer. Eluates were stored at −80 °C prior to analysis using RT-qPCR.

### 2.4. RT-qPCR of Hepatitis A and Hepatitis E Virus

Whole nucleic acid eluates were tested using RT-qPCR. For HAV, this was carried out using primers and probes according to the method described by Costafreda and colleagues (2006) [23]. For HEV, it was conducted according to the method outlined by Jothikumar and colleagues (2006) [24] using a modified probe [25]. RT-qPCRs were performed using 5 µL eluate in a 30 µL reaction including ROX and using TaqPath 1-Step RT-qPCR Master Mix CG (Applied Biosystems by Thermo Fisher Scientific, Schwerte, Germany).

### 2.5. LOD and LOQ Establishment

To establish the limits of detection and quantification for hepatitis A and E viruses in sewage, samples with known concentrations of the viruses obtained from the national reference laboratory were spiked into fresh pond water in serial dilutions. Spiked pond samples were extracted shortly afterward, or after freezing at −80 °C, and were thawed on ice until extraction. Results were compared to calculate limits of detection and quantification as well as retrieval rates.

### 2.6. Comparison of Fresh and Frozen Wastewater

To compare the effect of one freeze–thaw cycle, the viral load of samples at −80 °C was compared with that of freshly concentrated wastewater. In total, nine samples were analyzed for HEV. Four samples from the sampling site Schmidbartlanger and five from the WWTP Gut Grosslappen were selected and are shown in Supplementary Figure S1.

### 2.7. RT-qPCR of PMMoV

Pepper Mild Mottle Virus (PMMoV) was used as a calibrator to normalize viral loads of human fecal matter content. PMMoV is among the most universally shed viruses in human feces. This plant virus does not depend on active infection and is shed by humans after ingesting pepper-containing food. It is therefore established in WBE for normalization [26]. Quantification of PMMoV from purified wastewater samples was carried out using RT-qPCR as described by the manufacturer’s protocol using the kit “GoTaq Enviro PMMoV Quant Kit, Quasar 670” (Promega GmbH, Walldorf, Germany). In brief, 5 µL of a 1:10 sample dilution was added to 10 µL of Master Mix, 1 µL of primer/probe mix, 3.6 µL of nuclease-free water, and 0.4 µL of enzyme mix. The standard thermal cycle program with detection of fluorophore Quasar 670/Cy5 (Promega GmbH, Walldorf, Germany), provided by the kit, was used for 40 cycles.

### 2.8. Normalization with PMMoV

The sewage system in Munich was constructed in the late 1800s and is still in use today. Furthermore, new components have been incorporated. At the time of initial construction, combined sewage systems were the standard. Although the newer quarters in Munich are piped to new standards with separate pipes for household wastewater and rainwater, respectively, we observe a significant surge in wastewater flow, especially during periods of heavy rain, melting snow, or foreign water. Therefore, it is important in Munich to correct the targeted virus concentrations by normalizing to a constant fecal indicator for dilutions observed here. Mean PMMoV concentrations of each sample were determined separately, independent of the source, such as WWTP influx or Schmidbartlanger, regardless of whether the sample was worked up freshly or after a freeze–thaw cycle. Rain events were thus considered for normalization. This value was used to quantify the absolute concentrations of HAV and HEV in wastewater.

### 2.9. Notification Data

Official notification data (German Infection Protection Act, IfSG § 7) for hepatitis A and hepatitis E infections were obtained weekly for the City of Munich from the website of the Robert Koch Institute, Berlin [15]. In the law, § 7 lists the specifics of the notification duties regarding laboratory-confirmed cases of notifiable diseases. The Munich Health Department (GSR) kindly provided detailed notification data for the sewage catchment area of Schmidbartlanger using zip codes covering the area. These data include residents in the area of Schmidbartlanger who tested positive via rapid test and/or PCR test and are reported to the Health Authority.

### 2.10. Statistical Analysis

To detect any evidence of seasonality in the wastewater concentration data, we conducted one-way ANOVA tests and Kruskal–Wallis tests by rank to compare the normalized hepatitis A and E concentrations in samples collected in spring (March through May) versus summer (June through August) versus autumn (September through November) versus winter (December through February).

To detect evidence of differences in hepatitis A or E virus prevalence by lockdown status, Welch’s *t*-tests and Wilcoxon rank sum tests were conducted comparing the normalized hepatitis A and E virus concentrations in samples collected while lockdown restrictions were in place in Bavaria (16 March 2020 to 16 June 2020, 9 December 2020 to 6 June 2021, and 11 November 2021 to 11 May 2022) versus all other time periods. To test the sensitivity of this analysis to different definitions of “lockdown,” the same statistical methods were used to compare samples collected before versus after 3 April 2022. On this date, the majority of COVID-19 regulations, including the closure of schools and restaurants, as well as travel bans, were lifted in Bavaria, while smaller precautionary regulations, such as the wearing of masks, were maintained. This same set of analyses was also performed on the weekly case notification data for Munich and its surrounding areas for 2020 through 2023. Finally, the correlations between the wastewater concentration data and the OxCGRT project’s COVID-19 Stringency Index for Germany were assessed using Pearson and Spearman correlation coefficients [27,28].

Potential correlations between wastewater concentration data and the corresponding notification data for each virus, summarized by week and month, were assessed using Spearman and Pearson correlation coefficients.

## 3. Results

The primary objective of this study was to evaluate the effectiveness of WBE in monitoring HAV and HEV circulation, and to determine whether the impact of SARS-CoV-2 lockdown measures can be quantified on these infections. Between July 2020 and November 2023, we collected 111 untreated wastewater samples from the Schmidbartlanger sampling site in northern Munich (Figure 2). Additionally, from November 2021 to November 2023, 75 twenty-four-hour composite wastewater samples were collected at the Gut Grosslappen treatment plant in Munich. All samples were tested for HAV and HEV, enabling an evaluation of WBE’s effectiveness in detecting these pathogens and identifying any changes associated with the lockdown and post-lockdown periods.

**Figure 3:**
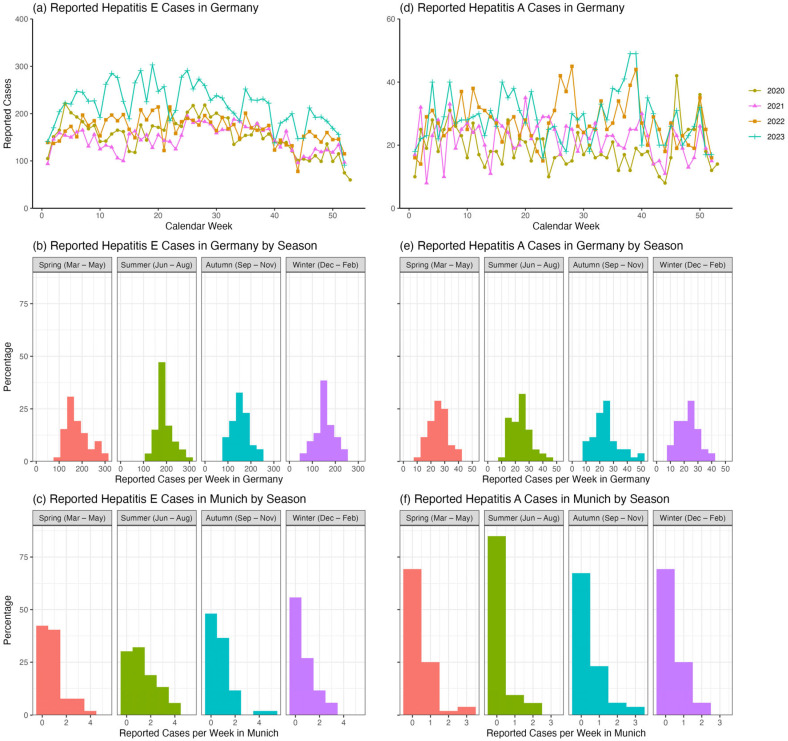
Reported cases of hepatitis A and E infections in Germany between 2020 and 2023. The left column shows trends in the number of HEV cases reported each week in Germany between 2020 and 2023, with case numbers plotted over time in Panel (**a**) (Table S1), by season for all of Germany in Panel (**b**), and by season for Munich only in Panel (**c**). During the years with lockdown periods (2020–2022), we generally saw lower numbers of reported cases than in 2023. Throughout each year, there is a noticeable seasonal pattern: Reported cases tend to drop towards the end of each year and rise in spring/summer. The right column shows the corresponding trends for Hepatitis A, with case numbers plotted over time in Panel (**d**) (Table S2), by season for all of Germany in Panel (**e**), and by season for Munich only in Panel (**f**). The colors in (**b**,**c**,**e**,**f**) represent the different seasons. Red indicates spring, green summer, blue autumn, and purple winter. See Supplementary Tables S1 and S2 for detailed numbers [15].

Although HEV and HAV are known to be significantly underreported, analyzing their reported case numbers provides some insights. The number of reported HEV cases in Germany appears to be lower between 2020 and 2022, when COVID-related lockdown measures were in place, compared to the post-pandemic year of 2023 (Figure 3a). Reported HEV cases evince a seasonal pattern both throughout Germany (Figure 3b; ANOVA: *p* < 0.001 and Kruskal–Wallis: *p* < 0.001) and in Munich only (Figure 3c; ANOVA: *p =* 0.01 and Kruskal–Wallis: *p =* 0.01), with fewer cases reported during the autumn/winter months at the beginning and end of each year. Specifically, in Munich, pairwise *t*-tests and Wilcoxon tests indicated that the summer average of 1.32 (SD: 1.20) HEV cases per week was higher than the autumn average of 0.77 (SD: 1.02) cases per week (*t*-test: *p =* 0.04; Wilcoxon: *p =* 0.06) and higher than the winter average of 0.67 (SD: 0.90) cases per week (*t*-test: *p* = 0.01; Wilcoxon: *p* = 0.02), but not higher than the spring average of 0.87 (SD: 0.99) cases per week (*t*-test: *p* = 0.15; Wilcoxon: *p* = 0.27). In contrast, reported HAV cases in Germany appear to be only slightly elevated in 2023, when there were no lockdown restrictions (Figure 3d). There were also no significant differences in weekly reported HAV cases by season across Germany (Figure 3e; ANOVA: *p* = 0.39 and Kruskal–Wallis: *p* = 0.27) or in Munich only (Figure 3f; ANOVA: *p* = 0.24 and Kruskal–Wallis: *p* = 0.17).

### 3.1. Persistent Detection of Hepatitis E Virus

**Figure 4:**
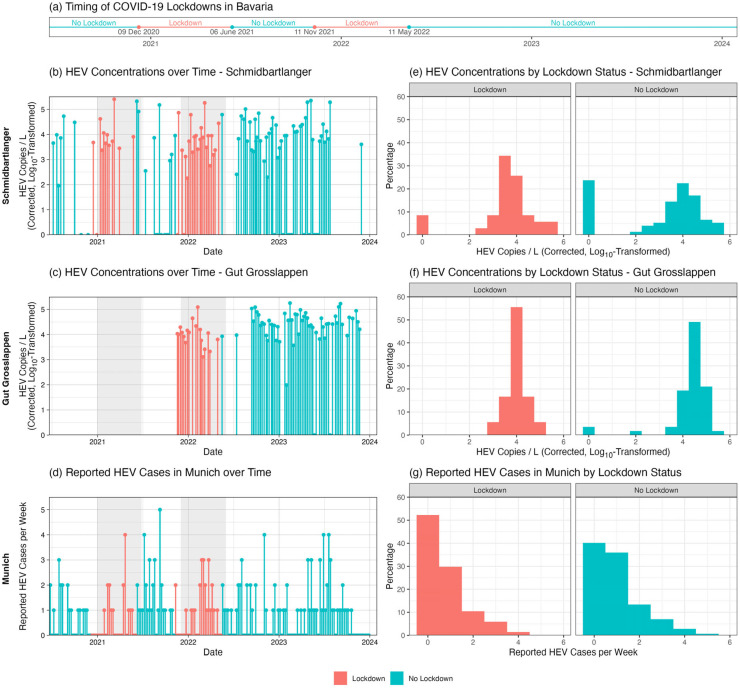
HEV concentrations by lockdown status. Lockdown periods in the state of Bavaria are visualized in a timeline (**a**). The left column shows the normalized HEV concentration over time for Schmidbartlanger (**b**) and Gut Grosslappen (**c**), as well as the weekly reported HEV cases in Munich (**d**), with lockdown periods marked in red and non-lockdown periods in blue. The gray-shaded regions indicate the lockdown periods shifted forward by three weeks, which is the estimated time from HEV infection to the start of shedding. Sampling at Schmidbartlanger was conducted between July 2020 and November 2023, and at the WWTP Gut Grosslappen between November 2022 and November 2023. The right column shows the distribution of normalized HEV concentrations by dichotomized lockdown status for Schmidbartlanger (**e**) and Gut Grosslappen (**f**), as well as the distribution of weekly reported HEV cases by dichotomized lockdown status (**g**).

To assess the applicability of WBE to Munich, we evaluated the detectability of HEV in wastewater samples. Therefore, we measured HEV viral loads in collected samples using RT-qPCR. Data were normalized using the viral loads of Pepper Mild Mottle Virus (PMMoV) obtained from the same samples. We observed consistently high concentrations of HEV genome equivalents in the range of 9.6 × 10^1^ to 1.8 × 10^5^ copies/L, an average of 3.6 × 10^4^ copies/L (±SD 3.8 × 10^4^), and a median of 2.4 × 10^4^ in the 24 h compound sample (Figure 4). The qualified spot sample harbors greater variability, most likely due to overrepresentation of single shedding events related to the respective sample. Here, the range of HEV genome equivalents was 9.0 × 10^1^ to 2.5 × 10^5^ copies/L, a mean value of 3.0 × 10^4^ copies/L (±SD 5.2 × 10^4^), and a median of 8.0 × 10^3^. This shows that summary statistics for Schmidbartlanger diverge from those for the 24 h compound sample at Gut Grosslappen, with the range being wider and the standard deviation being higher, but the median being lower.

**Figure 5:**
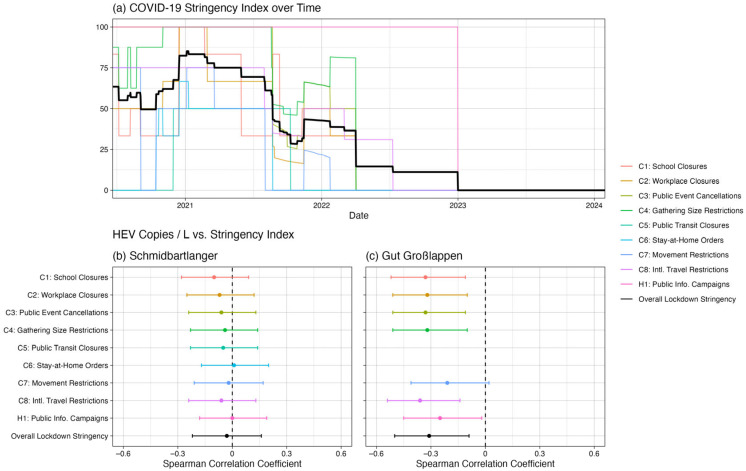
Correlations between HEV concentrations and lockdown stringency. Spearman correlation coefficients (with 95% confidence intervals) are plotted for the correlations between the overall COVID-19 Stringency Index (**a**), as well as its individual components, and the normalized HEV concentrations for Schmidbartlanger (**b**) and Gut Grosslappen (**c**). Dashed lines denote a Spearman correlation coefficient of zero.

Statistical analysis revealed no significant differences in HEV concentration by season for either Schmidbartlanger (ANOVA: *p* = 0.05; Kruskal–Wallis: *p* = 0.18) or Gut Grosslappen (ANOVA: *p* = 0.48; Kruskal–Wallis: *p* = 0.26). Analysis by lockdown status revealed higher HEV concentrations on average for Gut Grosslappen during no-lockdown compared to lockdown periods. The mean normalized HEV concentration was 1.8 × 10^4^ (SD: 2.8 × 10^4^) copies/L across lockdown periods in Bavaria, compared to 4.0 × 10^4^ (SD: 3.9 × 10^4^) copies/L across no-lockdown periods (*t*-test: *p* = 0.01; Wilcoxon: *p* = 0.002) (Figure 4c,f). Furthermore, there was a Pearson correlation of −0.25 (*p* = 0.03) and a Spearman correlation of −0.31 (*p* = 0.01) between the COVID-19 Stringency Index [28] and the HEV wastewater data for Gut Grosslappen, suggesting that HEV concentrations were generally higher when lockdown measures were less strict (Figure 5). This result was essentially unchanged if the index components not directly related to everyday mobility were removed (i.e., if the components “Public information campaign” and “Restrictions on international travel” were removed, leaving “School closures,” “Workplace closures,” “Public event cancellations, “Gathering size restrictions,” “Public transit closures,” “Stay-at-home requirements,” and “Restrictions on internal movement”).

As shown in Figure 4b,e, this pattern of differences by lockdown status did not hold for Schmidbartlanger, where the mean HEV concentration was roughly the same regardless of lockdown status: 2.2 × 10^4^ (SD: 5.1 × 10^4^) copies/L during lockdown periods and 2.5 × 10^4^ (SD: 4.9 × 10^4^) copies/L otherwise (*t*-test: *p* = 0.74; Wilcoxon: *p* = 1.00). The Pearson correlation (r = 0.04, *p* = 0.70) and Spearman correlation (ρ = −0.03, *p* = 0.75) between the Schmidbartlanger wastewater data and the COVID-19 Stringency Index [27,28] were negligible (Figure 5). Reported cases per week showed no significant difference between lockdown (mean = 0.75, SD: 0.98) and no-lockdown (mean = 0.99, SD: 1.09) periods (*t*-test: *p* = 0.11; Wilcoxon: *p* = 0.10) (Figure 4d,g). All results were very similar for our alternative definition of lockdown (before vs. after 3 April 2022).

**Figure 6:**
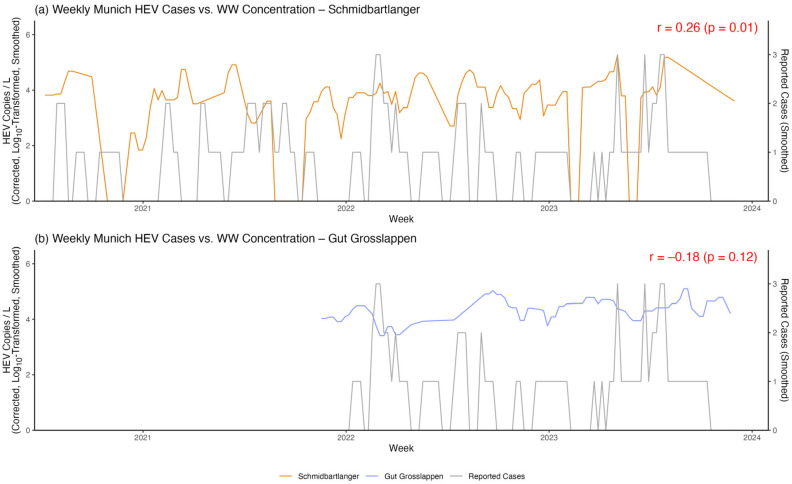
Cross-correlation between HEV wastewater concentrations and reported cases. Smoothed HEV concentrations over time for Schmidbartlanger (**a**) and Gut Grosslappen (**b**) are plotted here along with the corresponding reported HEV cases in Munich.

Overall, HEV notification data and wastewater concentrations were fairly evenly distributed over time. There was a small but significant positive correlation between the mean HEV concentration per week measured at Schmidbartlanger and the corresponding total number of cases in Munich, with r = 0.26 (*p* = 0.01), as shown in Figure 6a. For Gut Grosslappen, statistically significant correlations between wastewater measurements and cases were not observed, with r = −0.18 (*p* = 0.12), as shown in Figure 6b.

### 3.2. Sporadic Detection of Hepatitis A Virus

We also conducted research on HAV in wastewater samples in Munich. Viral load was measured using RT-qPCR, and normalization was also conducted with PMMoV. We observed HAV genome copies below the detection range in 92.5% (172/186) of the samples. If positive, both sampling sites showed considerable variability with values between 4.6 × 10^1^ and 2.4 × 10^3^ copies/L in the WWTP compound sample.

Sampling occurred weekly or bi-weekly, setting adequate time resolution for HAV, which is shed for several weeks during acute infection. We should detect several consecutive positive samples if enough infected subjects live in the sampled catchment area (Figure 2), and the concentration is above the detection limit. We observe several spikes indicating single events of detection and most likely sporadic detection of single excretion events. From January to July 2022, there was a notable occurrence of successive positive values in Gut Grosslappen. This might indicate a certain small outbreak in its catchment area. This could only be detected once in Schmidbartlanger, even on the same date. The high concentration of HAV in the Schmidbartlanger spot sample, 2.9 × 10^2^ copies/L, shows the dilution artifact once it reaches the WWTP, where the concentration is only 7.6 × 10^1^ copies/L.

Overall, the intervals with travel restrictions (2020–2021) do not show fewer notified cases than in 2022, when foreign travel was still lower than in pre-pandemic times, or in 2023, when it reached pre-pandemic values. Indeed, 2023 even seems to have a trend towards fewer detections in the wastewater and fewer cases in the notification data.

Statistical analysis revealed no significant differences in HAV concentration or reported cases by either season or lockdown status. There were no statistically significant differences in HAV concentration by season for either Schmidbartlanger (ANOVA: *p* = 0.09; Kruskal–Wallis: *p* = 0.43) or Gut Grosslappen (ANOVA: *p* = 0.33; Kruskal–Wallis: *p* = 0.28). For Schmidbartlanger, the mean normalized HAV concentration during lockdown periods, 0.8 × 10^1^ (SD: 5.0 × 10^1^) copies/L, was not significantly different from the concentration during no-lockdown periods, 4.1 × 10^1^ (SD: 2.5 × 10^2^) copies/L (*t*-test: *p* = 0.27; Wilcoxon; *p* = 0.78). Similarly, for Gut Grosslappen, the mean normalized HAV concentration during lockdown periods, 2.1 × 10^2^ (SD: 5.6 × 10^2^) copies/L, was not significantly different from the concentration during no-lockdown periods, 2.0 × 10^1^ (SD: 1.1 × 10^2^) copies/L (*t*-test: *p* = 0.18; Wilcoxon: *p* < 0.001). Mean HAV cases during lockdown periods, 0.39 (SD: 0.72) cases per week, were not significantly different from mean cases during non-lockdown periods, 0.35 (SD: 0.64) cases per week (*t*-test: *p* = 0.68; Wilcoxon: *p* = 0.78).

Correlation analysis of reported HAV cases versus wastewater concentrations was generally unrevealing. For both sampling locations, there were no clear correlations between the mean HAV concentration over time and the corresponding total number of cases when data were aggregated by month (r = −0.13 and ρ = 0.16 for Schmidbartlanger; r = 0.17 and ρ = 0.43 for Gut Grosslappen) or by week (r = −0.09 and ρ = −0.14 for Schmidbartlanger; r = −0.06 and ρ = −0.08 for Gut Grosslappen). Due to the low number of HAV detections in Munich sewage, overall correlations are difficult to interpret.

## 4. Discussion

We present a three-year wastewater sampling study for HAV and HEV, demonstrating the feasibility of tracking viral loads in both newly and previously collected deeply frozen samples. This study period encompassed SARS-CoV-2 lockdowns, offering a natural experiment to assess how contact and transmission reduction measures influenced the transmission of fecal–oral diseases hepatitis A and E. While HAV concentrations in wastewater were predominantly low and sporadic—likely reflecting a small number of infections—HEV concentrations remained high, underscoring the virus’s pervasive circulation in the study population.

As Munich is a non-endemic region for HAV, generally low concentrations in wastewater were anticipated. Despite sporadic detection, no direct correlation was found between wastewater and notification data. Rather than invalidating WBE, this finding suggests that longitudinal monitoring at a wastewater treatment plant’s inflow is more effective for larger outbreaks (e.g., foodborne clusters). Nevertheless, sampling such large areas is not suitable for detecting single sporadic cases due to the occurrence of dilution artifacts. This phenomenon appears to be consistent across both sampled regions. Interestingly, a cluster of positive samples was detected between January and July 2022 in the WWTP that could only be detected once in Schmidbartlanger due to the difference in the catchment area. Hepatitis A is considered a travel-related disease, which, due to its long incubation time and slow recovery, is mostly acquired and imported during international travel to high-risk countries. Looking at the travel restrictions imposed on the German population in 2020 and 2021, we would expect a great reduction in cases during these years. However, such a decline was not observed. Instead, a downward trend continued in 2023, following the end of restrictions. Notification numbers and wastewater concentrations continued to decrease further as international travel returned to pre-pandemic levels. This raises questions about the significance of travel-related HAV cases versus potentially dominant local transmission, already suggested by RKI data [29]. This observation might be caused by high vaccination rates or reduced exposure to HAV in travel destinations. Additionally, reports about contaminated frozen berries sold in Europe [30] and strawberry cake in Germany [17] solidify the case for local foodborne transmission chains. This provides an argument for developing an early warning system based on wastewater that could detect larger food-related outbreaks. Broader vaccination recommendations, not limited to travelers to high-risk areas, should be considered if local foodborne transmission already accounts for a larger share of infections than international travel.

For HEV, high concentrations were surprisingly abundant throughout the whole observation period. Given the seroconversion and blood donor data collected in Germany [12], it is expected that roughly one out of 1240–1474 inhabitants is shedding HEV at any given point, resulting in about 1000 individuals being HEV-positive at each time point in Munich [31,32]. Due to extended excretion periods even in asymptomatic cases, high viral loads in sewage are expected. Notification data for HEV infections is clearly dominated by symptomatic individuals. Despite the existence of a legal notification basis, subclinical (re-)infections will not be tested or reported, resulting in a large number of undetected cases. Our study demonstrates that WBE can confirm the continuous infection cycle during the lockdown periods in Munich. This dataset may be regarded as a unique “natural experiment” offering a valuable opportunity to identify potential risk factors associated with HEV infection. We detected a reduction in viral load but not in notification numbers during lockdown interventions, including closure of schools, limitation of personal contacts, social distancing, and closure of hospitality venues offering dine-in, food pick-up, or delivery options. It is hypothesized that the transmission of HEV in Central Europe occurs primarily via contaminated food, predominantly undercooked pork-containing products, mainly distributed via supermarkets. Nevertheless, definite proof remains elusive at this time. In addition, significant numbers of secondary cases exist in the households of HEV-excreting subjects. Given the evidence presented here, we conclude that the measures taken during the lockdown periods in Munich indeed influenced the ongoing transmission of HEV in the population, with drastic measures taken. Most (re-)infections appear to occur as a result of foods regularly sold by supermarkets and butcheries, so person-to-person contact or hospitality venues—offering dine-in, food pick-up, or delivery options—or both play a significant role in spreading the disease in Munich.

The outcomes of our study stand in notable contrast to those of a previous study from Spain [8]. While there was only a single finding of HEV in wastewater out of 106 samples reported, our analysis identified HEV in almost every sample from the WWTP and in most Schmidbartlanger samples. As notification numbers of HEV-infected individuals in Munich were also low, and pork meat is consumed in both countries, these findings are surprising.

This study has a few limitations. First, the samples were not taken daily, but only weekly or bi-weekly. This results in fewer data points and increased possible variability due to the lack of averaging over several days. However, as shedding is prolonged over a period of several weeks, the impact is not dramatic. At the beginning of the study, sampling at the WWTP inflow was not conducted. Consequently, there are no comparative data available for this time period. The WWTP inflow was not chosen in March 2020, as the decay for initial SARS-CoV-2 in sewage was suspected to be relatively rapid, so that only sampling spots with less than four hours of flow time were chosen. HAV and HEV are stable enough—there is no significant negative effect on the viral loads due to longer flow times.

Second, we compare qualified spot samples with 24 h compound samples, which could clearly introduce additional variability. We observe that there is no major difference, as the catchment area of the sampling site Schmidbartlanger is also sufficiently large for the sampling of HEV to deliver almost constant concentrations. In the case of HAV, sporadic detection was observed in both sample types. It would most likely benefit from even smaller areas sampled with compound samplers to ensure less dilution and time integration, and thus a constant detection of single-shedding patients. Fluctuating concentrations in sewage are caused by discontinuous viral shedding from individuals. Our sampling strategy is incapable of elucidating this effect. For HEV, this is not considered a significant matter because of the high number of infected individuals (estimated to be around 1000 in the city of Munich at any given time point) and the large catchment areas of our study. In the case of HAV, this effect will be one of the most important sources of variability in our data. We mitigated dilution artifacts, which occur due to rain or melting snow, by standardization using PMMoV loads. Still, dilutions might shift the viral load below the detection limit, so that it can no longer be corrected with the determined dilution factor.

We used deeply frozen samples and fresh samples. To compare these results, we determined the viral loads of nine samples in their fresh and frozen states. The raw data from the evaluation are displayed in Figure S1. In certain samples, the viral loads for HEV are slightly lower than in freshly concentrated and extracted samples, despite using the double volume to mitigate this effect. With this finding, it is possible that some of the deeply frozen samples show lower viral loads than in reality, or are even below the detection limit, but the effect is overall much less pronounced than with more fragile pathogens such as SARS-CoV-2.

Notification data might also have shortcomings. We can be confident that all laboratory-confirmed cases will actually be notified, as there are well-established notification systems in place. However, by no means will all patients receive a diagnosis; asymptomatic or oligosymptomatic individuals are especially unlikely to be tested for acute hepatitis and thus will not be reflected in the statistics. For HAV and especially for HEV, we can reasonably suspect that the majority of cases are never detected by a laboratory.

## 5. Conclusions

Reviewing the three years of wastewater sampling studied, HEV concentrations were surprisingly constant and high over time, proving considerable ongoing infection activity, leading to almost all sewage samples being positive for HEV. The social distancing measures, school closures, closures of hospitality venues offering dine-in, food pick-up, or a delivery option, and travel restrictions did change the wastewater concentrations, but not the notification numbers, during the lockdown periods. This proves the hypothesis that transmission occurs through regularly sold foods in combination with either person-to-person contact or hospitality venues offering dine-in, food pick-up, or delivery options, or both. This is a factor that contributes to the increase in HEV infections in Munich. For HAV, we observed surprisingly few positive samples. Although the disease is thought to be mainly travel-related, there was neither an increasing concentration of HAV in the sewage nor in notified cases at the end of 2022 and 2023, when international travel resumed after lifting COVID-19-related restrictions. This finding indicates that domestic transmission, potentially through contaminated food, may be as, or even more, relevant in causing the sporadic cases observed here. Moreover, the findings indicate that to identify sporadic HAV cases, it is necessary to investigate smaller catchment areas with compound samplers, where the sewer system is less diluted. Conversely, WWTP inflow is only suitable for picking up larger case clusters due to the combination of dilution artifacts and the low incidence of the virus.

## Data Availability

Data on GI-infections in Germany are openly available in a publicly accessible at https://survstat.rki.de/ repository, accessed on 15 March 2025. Data on HAV and HEV infections presented here are available on request from GSR Munich and are listed in the Supplementary Information Tables S1 and S2. The original contributions presented in this study are included in the article. Further inquiries can be directed to the corresponding author.

## Supplementary Materials

The following supporting information can be downloaded at https://www.mdpi.com/article/10.3390/microorganisms13102379/s1, Table S1: Table of reported Hepatitis A cases in the City of Munich according to IfsG § 7 between 2020–2023; Table S2: Table of reported hepatitis E cases in the City of Munich according to IfsG § 7 between 2020–2023; Figure S1: Comparison of viral loads of hepatitis E virus in fresh and frozen wastewater.

## Abbreviations

The following abbreviations are used in this manuscript:

1 h	1 hour
24 h	24 hour(s)
ANOVA	Analysis of variance
Copies/L	Copies per liter
COVID-19	Coronavirus disease of 2019
GI	Gastro-intestinal
HAV	Hepatitis A virus
HEV	Hepatitis E virus
IfsG	Infektionsschutzgesetz (German Infection Protection Act)
LoD	Limit of detection
LoQ	Limit of quantification
MSE	Munich Sewer Authority (Münchner Stadtentwässerung)
PMMoV	Pepper mild mottle virus
RNA	Ribonucleic acid
RT-qPCR	Reverse transcription—quantitative polymerase chain reaction
SARS-CoV-2	Severe acute respiratory syndrome coronavirus 2
SD	Standard deviation
WBE	Wastewater-based epidemiology
WW	Wastewater
WWTP	Wastewater treatment plant
