# Supplementary material for: Continuous Circulation of Hepatitis E and A Viruses During COVID-19 Pandemic Lockdowns in Munich, Germany—Experience from Three Years of Wastewater Surveillance
Source: Microorganisms. 2025 Oct 15;13(10):2379. doi: 10.3390/microorganisms13102379 (PMC12566045; doi:10.3390/microorganisms13102379)
Supplement: Supplementary file 1 [file microorganisms-13-02379-s001.zip › microorganisms-3878931-supplementary.pdf]

Supplementary Information

Continuous circulation of hepatitis E and A virus during COVID-19 pandemic lockdowns in Munich, Germany – experience from three years of wastewater surveillance

Jasmin Javanmardi[1*], Mathias Schemmerer[2], Karina Wallrafen-Sam[3], Jessica Neusser[1,4], Raquel Rubio-Acero[1], Michael Hoelscher[1,5,6,7], Thomas Kletke[8], Bernhard Boehm[8], Michael Schneider[8], Elisabeth Waldeck[9], Martin Hoch[10], Merle M. Böhmer[10,11], Christof Geldmacher[1,5,6], Jan Hasenauer[3,12], Jürgen J. Wenzel[2], Andreas Wieser[1,5,6,13]

[1] Institute of Infectious Diseases and Tropical Medicine, LMU University Hospital, LMU Munich, 80802 Munich, Germany; jasmin.javanmardi@med.uni-muenchen.de (J.J.); jessica.neusser@lgl.bayern.de (J.N.); raquel.rubio@med.uni-muenchen.de (R.R.-A.); michael.hoelscher@med.uni-muenchen.de (M.H.); christof.geldmacher@med.uni-muenchen.de (C.G.)

[2] National Consultant Laboratory for HAV and HEV, Institute of Clinical Microbiology and Hygiene, University Medical Center Regensburg, 93053 Regensburg, Germany; mathias.schemmerer@klinik.uni-regensburg.de (M.S.); juergen.wenzel@klinik.uni-regensburg.de (J.J.W.)

[3] Life & Medical Sciences Institute (LIMES), Bonn Center for Mathematical Life Sciences, University of Bonn, 53115 Bonn, Germany; karina.wallrafen@uni-bonn.de (K.W.-S.); jan.hasenauer@uni-bonn.de (J.H.)

[4] Department Task Force Infectious Diseases, Bavarian Health and Food Safety Authority, 80636 Munich, Germany

[5] Immunology, Infection and Pandemic Research, Fraunhofer Institute for Translational Medicine and Pharmacology ITMP, 80799, Munich, Germany

[6] German Center for Infection Research (DZIF), Partner Site Munich, 80802 Munich, Germany

[7] Unit Global Health, Helmholtz Zentrum München, German Research Center for Environmental Health, 85764 Munich, Germany

[8] Munich Metropolitan Sewer Authority (MSE), 81671 Munich, Germany; thomas.kletke@muenchen.de (T.K.); bernhard.boehm@muenchen.de (B.B.); m.schneider@muenchen.de (M.S.)

[9] Public Health Department Munich (GSR), 80335 Munich, Germany; elisabeth.waldeck@muenchen.de (E.W.)

[10] Task Force for Infectious Diseases (GI-TFI), State Institute for Health II, Bavarian Health and Food Safety Authority (LGL), 80636 Munich, Germany; martin.hoch@lgl.bayern.de (M.H.); merle.boehmer@lgl.bayern.de (M.M.B.)

[11] Institute of Social Medicine and Health Systems Research, Otto-von-Guericke-University, 39120 Magdeburg, Germany

[12] Institute of Computational Biology, Helmholtz Zentrum München—German Research Center for Environmental Health, 80939 Neuherberg, Germany

[13] Max von Pettenkofer Institute of Hygiene and Medical Microbiology, Faculty of Medicine, LMU Munich, 80336 Munich, Germany

* Correspondence: wieser@mvp.lmu.de

1. Reported cases

1.1: Reported cases of Hepatitis A infections in Germany.

Table S1 shows the reported Hepatitis A cases in the city of Munich according to IfsG §7 during the years 2020-2023. In Figure 2f the numbers are visualized in a graph. Case numbers were kindly provided by the GSR Munich.

Table S1. Table of reported Hepatitis A cases in the City of Munich according to IfsG § 7 between 2020 - 2023. Data provided by the GSR Munich.

Calendar week	2020	2021	2022	2023
1	10	17	16	18
2	25	32	14	22
3	19	8	29	23
4	28	23	31	40
5	18	28	27	22
6	25	10	23	29
7	31	33	25	40
8	26	19	27	27
9	23	25	37	28
10	16	27	25	28
11	27	24	38	29
12	17	26	32	30
13	13	20	31	23
14	18	11	29	31
15	18	28	27	26
16	14	26	21	40
17	28	24	27	35
18	16	19	29	38
19	22	20	23	31
20	21	35	28	27
21	15	22	23	37
22	22	26	18	28
23	22	29	15	16
24	10	29	27	25
25	16	25	31	26
26	17	17	42	21
27	14	26	37	18
28	15	25	45	30
29	24	18	26	28
30	17	24	24	30
31	20	22	26	18
32	16	27	25	25
33	17	18	34	33
34	16	23	25	28
35	21	23	27	38
36	12	20	34	37
37	17	19	29	41
38	12	25	39	49
39	19	25	44	49
40	17	30	27	20
41	18	23	20	35
42	14	14	29	30

43	10	15	25	20
44	8	11	18	20
45	16	27	27	26
46	42	23	19	31
47	23	19	23	20
48	25	13	20	23
49	25	16	19	26
50	36	26	35	32
51	18	19	25	17

1.2: Reported cases of Hepatitis E infections in Germany.

Table S2 shows the reported Hepatitis E cases in the city of Munich according to IfsG §7 during the years 2020-2023. In Figure 2c the numbers are visualized in a graph. Case numbers were kindly provided by the GSR Munich.

Table S2. Table of reported Hepatitis E cases in the City of Munich according to IfsG § 7 between 2020 - 2023. Data provided by the GSR Munich.

Calendar week	2020	2021	2022	2023
1	105	94	139	141
2	151	146	137	170
3	165	159	142	204
4	221	154	163	222
5	202	150	173	220
6	193	161	151	247
7	183	165	197	245
8	168	131	175	226
9	175	156	185	227
10	141	125	153	192
11	142	133	187	262
12	157	129	197	285
13	165	106	185	276
14	162	100	198	226
15	120	157	167	189
16	118	163	149	265
17	176	144	208	291
18	144	155	187	225
19	174	128	207	303
20	171	155	214	247
21	165	143	122	257
22	208	141	214	185
23	179	125	158	207
24	173	154	183	277
25	203	195	190	291
26	217	181	186	252
27	192	185	176	273
28	218	183	196	259
29	193	178	182	228
30	201	159	164	238
31	192	166	192	233
32	191	166	168	212

33	135	188	177	201
34	145	184	148	185
35	154	172	201	252
36	155	169	170	229
37	179	179	165	228
38	147	164	167	231
39	157	168	175	222
40	142	143	123	138
41	138	129	144	180
42	139	163	134	187
43	121	122	132	200
44	101	96	78	147
45	104	109	152	148
46	100	106	162	212
47	111	125	152	192
48	99	119	140	193
49	136	123	160	184
50	99	118	145	169
51	115	134	146	156
52	75	97	115	91
53	60	..	..	..

2. Comparison of wastewater conditions

2.1: Comparison of viral loads of Hepatitis E virus in fresh and frozen wastewater.

Viral loads in freshly drawn wastewater were compared to wastewater with one freeze-thaw cycle (frozen) at -80 °C.

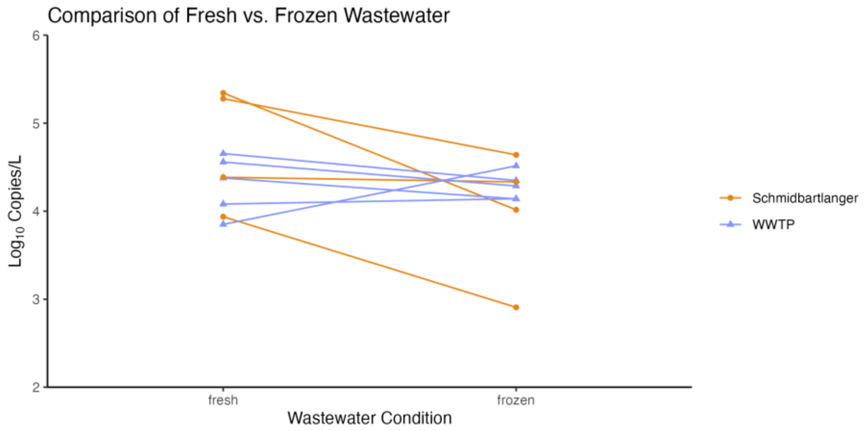

Figure S1. Comparison of viral loads of hepatitis E virus in fresh and frozen wastewater. Samples were concentrated as described in the manuscript section 2.3. Viral loads were estimated with RT-qPCR described in the manuscript section 2.7. Overall, there is not a concerning amount of loss of viral loads in frozen compared to freshly drawn samples.
